# A Comprehensive Review of Pathological Mechanisms and Natural Dietary Ingredients for the Management and Prevention of Sarcopenia

**DOI:** 10.3390/nu15112625

**Published:** 2023-06-03

**Authors:** Juhae Kim, Joo-Yeon Lee, Choon Young Kim

**Affiliations:** 1 Research Institute of Human Ecology, Yeungnam University, Gyeongsan 38541, Gyeongbuk, Republic of Korea; kimjh825@yu.ac.kr (J.K.); jooyeonlee@ynu.ac.kr (J.-Y.L.); 2 Department of Food and Nutrition, Yeungnam University, Gyeongsan 38541, Gyeongbuk, Republic of Korea

**Keywords:** sarcopenia, botanical extracts, marine extracts, bioactive compounds, probiotics, muscle, aging

## Abstract

Sarcopenia is characterized by an age-related loss of skeletal muscle mass and function and has been recognized as a clinical disease by the World Health Organization since 2016. Substantial evidence has suggested that dietary modification can be a feasible tool to combat sarcopenia. Among various natural dietary ingredients, the present study focused on botanical and marine extracts, phytochemicals, and probiotics. Aims of this review were (1) to provide basic concepts including the definition, diagnosis, prevalence, and adverse effects of sarcopenia, (2) to describe possible pathological mechanisms including protein homeostasis imbalance, inflammation, mitochondrial dysfunction, and satellite cells dysfunction, and (3) to analyze recent experimental studies reporting potential biological functions against sarcopenia. A recent literature review for dietary ingredients demonstrated that protein homeostasis is maintained via an increase in the PI3K/Akt pathway and/or a decrease in the ubiquitin–proteasome system. Regulation of inflammation has primarily targeted inhibition of NF-κB signaling. Elevated Pgc-1α or Pax7 expression reverses mitochondrial or satellite cell dysfunction. This review provides the current knowledge on dietary components with the potential to assist sarcopenia prevention and/or treatment. Further in-depth studies are required to elucidate the role of and develop various dietary materials for healthier aging, particularly concerning muscle health.

## 1. Introduction

**Figure 1:**
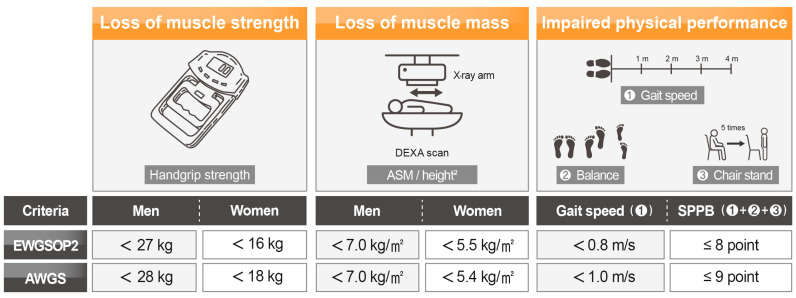
Criteria for diagnosis of sarcopenia. Abbreviations: AWGS, Asian Working Group for Sarcopenia; EWGSOP2, the European Working Group on Sarcopenia in Older People in 2019, SPPB, short physical performance battery.

Sarcopenia, a prevalent muscle disease in older adults, is characterized by a loss of muscle mass and strength, and performance and different criteria for diagnosis have been developed (Figure 1). Using the criteria of the European Working Group on Sarcopenia in Older People in 2019 (EWGSOP2), a muscle strength test is primary in predicting sarcopenia, and then, the muscle mass and physical performance are tested [1]. A handgrip strength of less than 27 and 16 kg for men and women, respectively, is used as a cut-off of the muscle strength index for sarcopenia. Appendicular skeletal muscle mass (ASM) assessed by dual-energy X-ray absorptiometry followed by adjustment for height (ASM/height^2^) is usually considered for muscle mass index, i.e., <7.0 kg/m^2^ in men and <5.5 kg/m^2^ in women are considered as sarcopenia. Low physical performance detected by a single cut-off value for gait speed <0.8 m/s measured over a distance of 4 m is widely used. Meanwhile, the cut-off points of each criterion by Asian Working Group for Sarcopenia (AWGS) are similar, although different from those by EWGSOP2: handgrip strength under 28 kg for men and 18 kg for women, muscle mass under 7.0 kg/m^2^ in men and less than 5.4 kg/m^2^ in women, and gait speed of less than 1.0 m/s are used as diagnostic criteria for sarcopenia [2]. In addition to the above, other cut-offs and measuring methods depending on the expert working group are known [1,2]. For example, short physical performance battery (SPPB), which consists of balance, chair stand, and gait speed test, is also used for diagnosing sarcopenia by measuring physical performance; it is less than eight points according to EWGSOP2 and nine points according to AWGS. Therefore, the prevalence of sarcopenia also depends on the cut-off type and varies widely. Using the EWGSOP2 definition and diagnosis, the prevalence of sarcopenia has been reported as 2 to 17% in subjects older than 60 years of age [3]. Sarcopenia prevalence was estimated as 41.0% by the AWGS criteria in individuals >65 years of age [4]. Moreover, the global population of more than 60-year-old adults, which was 1 billion in 2019, is predicted to expand to 1.4 billion by 2030 and 2.1 billion by 2050, worldwide [5]. Thus, with this growing aging population, sarcopenia is now considered a great public concern affecting individuals and the society.

**Figure 2:**
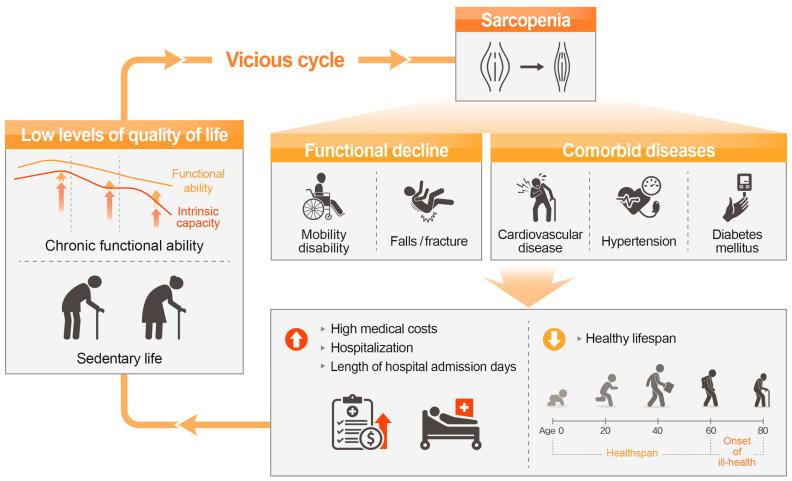
The vicious cycle of sarcopenia.

People with sarcopenia are at an increased risk of functional decline such as mobility disability [6] as well as comorbid diseases [7], resulting in increased medical expenses (Figure 2). Mobility refers to the ability of a person to move oneself in their environment, i.e., walking from one’s home to the neighborhood [8]. This mobility limitation is associated with an increased risk of falls [8]. In line with this, a recent meta-analysis showed a significant increase in the risk of falls indicated by pooled odd ratio of 1.69 (95% confidential intervals (CI): 1.43–2.00) in older adults with sarcopenia compared with that of older adults without sarcopenia [9]. Furthermore, a high prevalence or odds ratio of sarcopenia in individuals with comorbid diseases has been reported. Examples of diseases are cardiovascular diseases, hypertension, and diabetes mellitus, which have 31.4% of prevalence [7], 1.29 of odd ratio [10], and 2.09 of odd ratio [11] in patients with sarcopenia over those of non-sarcopenic subjects, respectively. With these adverse health effects, increased risk of hospitalization by pooled hazard ratio of 1.57 (95% CI: 1.26–1.94) in older adults with sarcopenia compared with that of non-sarcopenic older adults was recently reported [12]. Therefore, from a social point of view, sarcopenia is responsible for considerable healthcare expenditure. Medical costs attributable to sarcopenia were US $40.4 billion for individuals, with an average per person cost of US $260 [13]. In the UK, annual costs for muscle weakness in older people was estimated to be approximately ₤2.5 billion [14]. In addition, the proportion of years to be lived with disabilities and unhealthy life expectancy in sarcopenic older adults was increased by 6.6 times compared with that of non-sarcopenic older adults, as reported in a recent study of community-dwelling older adults aged >60 years [15]. Moreover, the association between severe sarcopenia and low quality of life, which was explained by sarcopenia-related disability, was observed in recent data on 14,585 people aged >65 years [16]. The negative health effects leading to comorbid diseases along with healthcare burden as well as low quality of life have resulted in sarcopenia being recognized as a disease and not as a simple symptom of normal aging. In this context, in 2016, sarcopenia was officially included in the International Classification of Disease, Tenth Revision, Clinical Modification (ICD–10–CM) [17]. In addition, sarcopenia received an ICD-10-CM (M62.5) code in the eighth revision of the Korean Classification of Diseases in 2021 [18]. Taken together, individual and social burdens increase the need for a sustainable preventive strategy aimed at preserving muscle health and function to reduce the sarcopenic population.

Several pharmacological candidates with mechanisms of action including hormone replacement, vitamin D replacement, and myostatin inhibition are under clinical evaluation [19]. However, mainstay suggestions for preventive approaches against sarcopenia are lifestyle modification, such as moderate exercise and a good quality diet. Although exercise is generally considered the primary strategy [20], the proportion of older adults meeting the physical activity recommendations ranged widely from 2.4 to 83.0% depending on studies [21]. Therefore, a dietary approach seems to be a visible action against sarcopenia. Foods are good sources of nutrients as well as bioactive compounds such as flavonoids, phenolic acid, and carotenoids. In particular, phytochemicals are known to regulate the same genes and pathways targeted by drugs [22]. Additionally, in recent times, probiotics have gained more attention as a potential strategy to prevent sarcopenia given that the gut–muscle axis plays an important role in regulating the onset and progression of age-related sarcopenia by its involvement in the pathophysiology of the disease, such as inflammation and protein homeostasis [23,24].

This review describes the current knowledge on dietary supplement candidates, which can be further developed as dietary supplements based on scientific evidence explained below, showing promising preventative or therapeutic effects on sarcopenia. Herein, the well-known pathophysiology of sarcopenia was first explained. Then, the results of previous studies on the effects of dietary factors such as botanical and marine extracts, phytochemicals, and probiotics on sarcopenia were summarized based on their mechanisms of action. Understanding the effects of these dietary factors on muscle health in experimental models can be utilized to develop dietary supplements or gain insights to identify a modifiable target for drug development in the near future.

## 2. Sarcopenia Pathophysiology

**Figure 3:**
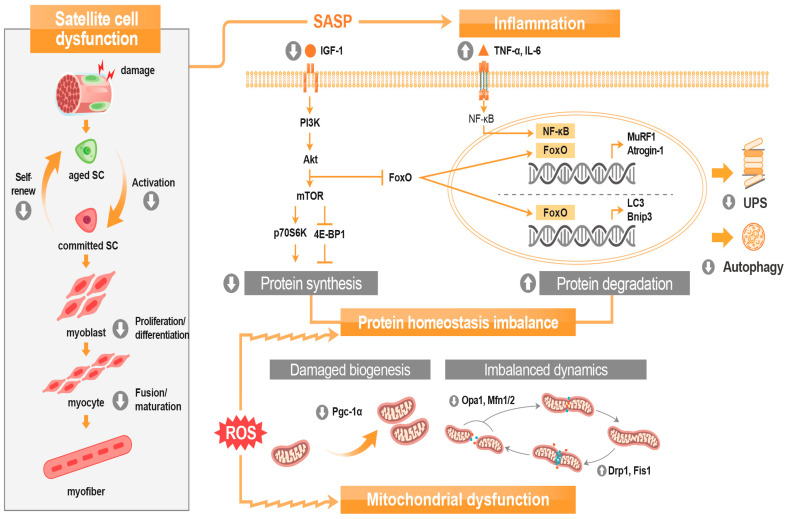
Overview of sarcopenia pathophysiology. Abbreviations: Akt, protein kinase B; Bnip3, Bcl-2 interacting protein 3; Drp1, dynamin-related protein 1; Fis1, fission 1 homolog protein; FoxO, forkhead box O; IGF-1, insulin-like growth factor-1; LC3, light chain 3; Mfn, mitofusin; mTOR, mammalian target of rapamycin; MuRF1, muscle RING-finger protein-1; NF-κB, nuclear factor kappa B; Opa1; optic atrophy-1; PI3K, phosphatidylinositol 3-kinase; Pgc-1α, proliferator-activated receptor γ coactivator-1 alpha; p70S6K, ribosomal protein S6 kinase; ROS, reactive oxygen species; SC, satellite cell; SASP, senescence-associated secretory phenotype; UPS, ubiquitin-proteasome system; 4E-BP1, 4E-binding protein 1.

The pathophysiology of sarcopenia has multifactorial causes. Some cellular and molecular mechanisms have been suggested to be involved that include protein homeostasis imbalance, inflammation, mitochondrial dysfunction, and satellite cell dysfunction (Figure 3). These factors do not independently cause sarcopenia but interact with each other to cause sarcopenia.

### 2.1. Protein Homeostasis Imbalance

The balance between protein synthesis and breakdown, also known as protein homeostasis, contributes to the maintenance of mass in skeletal muscle, the largest reservoir of amino acids in the body. Aging induces a negative balance between protein synthesis and degradation. Specifically, a reduction in protein synthesis was reported with advancing age. The rate of synthesis of mixed muscle proteins including myofibrillar, mitochondrial, and sarcoplasmic proteins was reduced by 30% with advancing age. Additionally, the synthesis rate of myosin heavy chain (MHC), the contractile protein, was reduced in older people (~77 years of age) [25]. Moreover, mitochondrial protein synthesis decreases with age. In a post-absorptive human study, older men (75 years of age) showed lower postprandial muscle protein synthesis rates than young men (22 years of age) by 16% [26]. Besides changes in muscle protein synthesis, age-related alteration in protein degradation have also been reported [27].

Protein synthesis and protein degradation are regulated by different pathways. A major regulatory signaling pathway for protein synthesis involves insulin-like growth factor-1 (IGF-1)—phosphatidylinositol-3-kinase/protein kinase B (PI3K/Akt)—the mammalian target of rapamycin (mTOR) complex 1 axis [28]. IGF-1 stimulates muscle cells to activate downstream targets, which are required for protein synthesis [29]. This hormone interacts with its tyrosine kinase receptor to phosphorylate insulin receptor substrate (IRS)-1 and activates PI3K/Akt signaling [28]. Thereafter, phosphorylated Akt activates mTOR, resulting in phosphorylation of ribosomal protein S6 kinase (p70S6K) and the 4E-binding protein 1 (4E-BP1), which promotes protein synthesis by activating ribosomal protein S6 and releasing eukaryotic translation initiation factor eIF-4E, respectively. The level of IGF-1, the main anabolic signal in skeletal muscle, decreases with age [30]. Therefore, muscle protein synthesis declines with age.

Major regulating pathways for protein degradation in the muscle are the ubiquitin–proteasome system (UPS) and autophagy–lysosome system. The UPS rapidly eliminates misfolded proteins and short-lived regulatory proteins by labeling them with ubiquitin and transmitting them to the proteasome where proteins are degraded. The proteolytic component in the pathway is the 26S proteasome, composed of proteolytic 20S core particles capped by one or two 19S regulatory particles [31]. In this pathway, proteins fated to be degraded are linked to a chain of ubiquitin molecules by a cascade of ubiquitin ligases (E1, E2, and E3). Major muscle-specific E3 ubiquitin ligases are muscle atrophy F-box (MAFbx/atrogin-1) and muscle RING-finger 1 (MuRF1) [32]. After ubiquitin molecules attach to proteins by atrogin-1 and MuRF1, the polyubiquitin-tagged proteins are fragmented into peptides and amino acids by the 26S proteasome. The autophagy–lysosomal system, the other degradation machinery, is complementary to UPS-mediated degradation. An internal acidic pH environment coupled with different hydrolases and proteases in lysosomes enables efficient degradation of misfolded and aggregated proteins not degradable by the UPS [33]. Among the three types of autophagy in mammalian cells, namely, chaperone-mediated autophagy, microautophagy, and macroautophagy, macroautophagy plays a major role in muscle protein degradation [27]. Macroautophagy is responsible for removing entire regions of the cytosol such as dysfunctional organelles and protein aggregates by the formation of a double membraned vesicle called a phagosome followed by its fusion with lysosomes where the cargo is normally degraded. The elongation and expansion of the double membrane is due to two ubiquitin-like proteins, autophagy-related 12 (Atg12) and LC3, a human homolog of yeast Atg8. Atg12 is conjugated to Atg5, mediated by Atg7 and Atg10, and then interacts with Atg16 in a non-covalent manner. Non-lipidemic LC3-I is converted to lipidemic LC3-II, which is an autophagosomal marker whose upregulated levels indicate upregulation of autophagy, which integrates into the autophagosomal membrane. Additionally, the accumulation of p62, a receptor for cargo destined to be degraded by autophagy is considered a sign of impaired autophagy [34]. In skeletal muscles, the forkhead box O3 (FoxO3) is the main controller of the transcription of autophagy-related genes, including *Bnip3* and *Beclin1*, as well as the aforementioned LC3, Atg4, Atg8, and Atg12 [35].

Several signaling pathways are interconnected in protein homeostasis. PI3K/Akt, induced by IGF-1 and enhances protein synthesis can also prevent protein degradation by phosphorylating and inactivating the FoxO transcription factor. The inactivated FoxO, which fails to translocate to the nucleus, prevents the upregulation of UPS genes including MuRF-1 and atrogin-1 in the skeletal muscle [36]. Myostatin, an autocrine and paracrine hormone produced by muscle cells, known to be a negative regulator of muscle mass, is also related to the degradation pathway. After myostatin binds to the activin type two receptor, downstream effector Smad2/3 is phosphorylated and activated. This phosphorylated Smad2/3 inhibits phosphorylation and activation of Akt leading to the reduction of FoxO phosphorylation. Unphosphorylated and, thereby, activated FoxO, as well as phosphorylated Smad2/3 can induce MuRF-1 and atrogin-1 expression, thereby inducing protein degradation [37]. In contrast, the inhibited Akt/mTOR pathway in response to myostatin also suppresses protein synthesis [38].

Several physiological changes during the aging process such as hormonal alteration and systemic inflammation have been demonstrated to affect either protein synthesis or protein degradation [39,40]. IGF-1 level was lower in the sarcopenic group than that in the non-sarcopenic group (98.53  ±  28.45 vs. 136.41  ±  48.95 ng/mL) in a cross-sectional study of more than 3000 subjects [41], which can impact protein homeostasis. The age-related decline of IGF-1 level, resulting from reduced liver IGF-1 production and the ability of skeletal muscle cells to produce IGF-1 locally is linked to protein synthesis reduction in older adults [42]. In addition, the salivary level of glucocorticoids, released from the adrenal cortex, which becomes dysfunctional in aging, increased in older adults with sarcopenia than in those without sarcopenia [43]. The increased levels of glucocorticoids inhibit protein synthesis and stimulate proteolysis in the skeletal muscle. Furthermore, increased expression of pro-inflammatory cytokines, such as tumor necrosis factor-alpha (TNF-α), play a role as a mediator for stimulating muscle atrophy signaling [40].

### 2.2. Age-Related Chronic Low-Grade Inflammation

The increase in inflammation during the aging process, also known as inflammaging, has been suggested as one of the significant contributors to sarcopenia [44,45]. Inflammaging is characterized as low-grade, chronic, and systemic inflammation without infection. This low-grade and chronic inflammation makes it possible to differentiate sarcopenia from cachexia, which is related to cancer-related inflammation-induced muscle wasting. Inflammaging is a consequence of immunosenescence and cellular senescence [46]. Immunosenescence, the decline in immune function induced by aging, is characterized by thymic atrophy, accumulation of senescent T-cells, and impaired innate immunity in natural killer cells, macrophages, and neutrophils [47]. The senescent cells, which are in a permanent state of cell cycle arrest are nonproliferating cells but they display a senescence-associated secretory phenotype (SASP). The SASP releases proinflammatory cytokines, including TNF-α, interleukin-6 (IL-6), and C-reactive protein (CRP), which are known to be involved in the pathophysiology of age-related sarcopenia. 

TNF-α, a proinflammatory cytokine whose levels are elevated in aged subjects, has been demonstrated to be involved in the progression of sarcopenia [48]. In a Chinese cohort study, high levels of TNF-α (>11.15 pg/mL) were associated with a 7.6-fold increased risk of sarcopenia [49]. A strong association between decline in grip strength and the TNF-α level was also demonstrated in a five-year study of change in thigh muscle area of 2177 participants aged 70–79 years [50]. Interestingly, pharmacological TNF-α blockade in 16–28-month-old mice prevented muscle atrophy and loss of type II fibers, accompanied by overall muscle function improvement [51]. IL-6, a proinflammatory cytokine secreted by many cells including monocytes and T lymphocytes is also associated with sarcopenia [48]. In a cross-sectional study of healthy older individuals, higher plasma levels of IL-6 and TNF-α were associated with lower muscle mass and lower muscle strength [52]. Additionally, significantly increased IL-6 (49.77 in sarcopenia vs. 39.72 pg/mL in control) levels have been reported in another cross-sectional study of 441 patients over >60 years of age [53]. The age-related increases in circulating IL-6 levels and their significant contribution to declines in skeletal muscle strength, quality, and function were also demonstrated in a randomized controlled clinical trial (RCT) with 99 mobility-limited older adults [54]. In addition, CRP produced by the liver has been reported to be significantly increased in the sarcopenia group compared to the control group based on a meta-analysis from 17 cross-sectional studies [44]. High CRP (>6.1 μg/mL) and IL-6 (>5 μg/mL) levels were associated with a two- to three-fold greater risk of losing muscle strength in a longitudinal three-year follow-up study conducted with 986 subjects with a mean age of 75 years [55].

The nuclear factor-kappa B (NF-κB) is the main regulator of inflammatory cytokine-mediated muscle atrophy. NF-κB comprises the subunits such as Rel/p65, c-Rel, and RelB which possess transactivation domains [56]. In an inactive state, NF-κB resides in the cytosol, tightly bound to the inhibitory protein IκB. In response to proinflammatory cytokine TNF-α, IκB kinase phosphorylates IκB and initiates IκB degradation via the ubiquitin–proteasome pathway, leaving NF-κB free and active; NF-κB then translocates to the nucleus and binds to promoter sequences at the κB domain [57]. Increased NF-κB signaling enhances inflammation by upregulating cytokine release and increases protein degradation by the UPS with upregulation of MuRF1 and atrogin-1 expression.

Meanwhile, the aging of skeletal muscle is possibly associated with increased nitration, a maker for oxidative stress, which can be increased with an inflammatory status [58,59]. The muscle is known to be vulnerable to oxidative stress and a key enzyme is the sarcoplasmic/endoplasmic-reticulum Ca^2+^-ATPase (SERCA) [60]. Modification of nitration in aged muscle is increased by three-fold with a 40% loss in SERCA activity. Therefore, based on the fact that the physiological role of SERCA is to mediate muscle relaxation, inhibition of SERCA by nitration is likely to explain the muscle relaxation disorder observed in older subjects [61]. Moreover, a human study showed that high circulatory markers of inflammation are associated with peripheral muscle fatigue manifested by slower muscle contraction and relaxation in hospitalized geriatric patients [62].

### 2.3. Mitochondrial Dysfunction

A genomic and proteomic profiling study showed that genes involved in mitochondrial energy metabolism pathway were downregulated in aged rats and a significant correlation between gene expression and sarcopenia was observed [63]. Decreased expression of genes encoding subunits of the mitochondrial electron transport chain was also reported in human aged muscle samples [64]. Thus, mitochondrial dysfunction is suggested as a pathophysiology of sarcopenia. Impaired mitochondrial biogenesis and mitochondrial dynamics have been proposed to cause mitochondrial dysfunction in sarcopenic muscles [65].

#### 2.3.1. Reduction of Mitochondrial Biogenesis

Mitochondrial biogenesis is a process that generates new mitochondria, with an increase in the number and density of mitochondria. In the muscle, which has a high metabolic rate, mitochondrial biogenesis is particularly important for producing ATP, which can facilitate muscle contraction and impact physical performance [66].

The process of mitochondrial biogenesis engages coordination between mitochondrial and nuclear genomes because mitochondrial proteins are encoded by nuclear and mitochondrial genomes. Although most of the proteins involved in mitochondrial genesis are encoded by the nuclear genome, a small number of crucial subunits of the electron transport chain complexes are encoded by mitochondrial DNA (mtDNA) [67]. In mtDNA transcription and translation, proliferator-activated receptor γ coactivator-1 alpha (Pgc-1α) is the master regulator of mitochondrial biogenesis. The Pgc-1α activation by either phosphorylation or deacetylation initiates the mitochondrial biogenesis, followed by stimulation of a series of nuclear transcription factors, such as nuclear respiratory factor-1 (Nrf-1), Nrf-2, and estrogen-related receptor alpha (ERRα). Once activated, Nrf-1/2 cofactors increase the expression of mitochondrial transcription factor A (Tfam), the final effect of mtDNA transcription and replication [68]. For proteins encoded by nuclear DNA (nDNA), all steps including transcription, translation, and preprotein synthesis occur in the cytoplasm. Translocase TIM23 is involved in directing the signal of preproteins toward the mitochondrial matrix. The nDNA as well as mtDNA encode proteins, thereby increasing mitochondrial biogenesis [68]. 

A decrease in mitochondrial biogenesis in aging as well as in sarcopenia have been reported in human as well as in vivo studies. Compared to those in young participants (22–24 years old), 50% of expression levels of key metabolic regulators of mitochondrial biogenesis, including sirtuin 3 (Sirt3) and Pgc-1α in the skeletal muscle of aged subjects (75–81 years old) was observed [69]. Moreover, individuals with sarcopenia exhibited reduced expression levels of Pgc-1α, ERRα, and other coactivators, such as Nrf-1 and Nrf-2, compared with those in individuals without sarcopenia [70]. In senescence-accelerated mouse prone-8 (SAMP8) mice, a well-studied murine model for age-related disease exhibiting typical features of skeletal muscle senescence, downregulation of Pgc-1α, Nrf-1, and Tfam during the onset and development of sarcopenia was observed [71].

#### 2.3.2. Dysregulation of Mitochondrial Dynamics

Mitochondrial function is regulated by mitochondrial dynamics, and the processes are composed of the fusion and fission of mitochondria [72]. Due to mixed contents in the mitochondria via fusion and fission, a homogenous and healthy mitochondrial population can be maintained. By mitochondrial fusion, damaged mitochondria are fused into healthy mitochondria, resulting in the complementation of damaged features. Meanwhile, damaged parts from healthy mitochondria can be isolated and discarded via mitophagy [73]. Mitochondrial functions such as mtDNA stability, respiratory capacity, and mitophagy are linked to normal mitochondrial dynamics [72].

Mitochondrial proteins involved in fusion and fission are well characterized. Mitochondrial fusion is a two-step process including outer mitochondrial membrane (OMM) fusion and inner mitochondrial membrane (IMM) fusion. Several GTPase proteins mediate the fusion process. They are mitofusin 1 (Mfn1) and Mfn2 for OMM fusion, and optic atrophy-1 (Opa1) for IMM fusion. In mitochondrial fission, dynamin-related protein 1 (Drp1) binds to mitochondrial outer membrane proteins, which are four Drp1 receptors. They are fission 1 homolog protein (Fis1), mitochondrial fission factor (Mff), mitochondrial dynamics protein of 49 kDa (Mid49), and mitochondrial dynamic protein of 51 kDa/mitochondrial elongation factor1 (Mid51/Mief1). This recruitment of Drp1 from the cytosol to mitochondria is required for the mitochondrial fission process [73].

Previous studies have reported that mitochondrial dynamics are dysregulated in the skeletal muscle of individuals with sarcopenia. Mediators involved in mitochondrial dynamics are decreased or deficient in aged muscles. For instance, aged (35-month-old) rats possessed smaller mitochondria and exhibited increased levels of fission proteins including Drp1 and Fis1, by approximately two–three folds compared to young (5-month-old) rats [74]. In a human biopsy study, the protein expression levels of mitochondrial fusion factor Mfn2 were revealed to be significantly reduced in older individuals with sarcopenia than those in control individuals [75].

### 2.4. Satellite Cell Dysfunction

Satellite cells (SCs), skeletal muscle stem cells, regenerate repeatedly throughout life and preserve skeletal muscle integrity contributing to muscle mass and muscle regeneration after injury. Thus, SC dysfunction has been suggested to be closely related to the onset and progression of sarcopenia [76,77].

SCs are normally quiescent and are present peripheral to the myofiber and underneath the basal lamina. Upon injury, such as muscle damage or trauma, SCs are driven out of their quiescent state, become activated, and regenerate muscle. The regeneration of muscle consists of several steps. The committed SCs are activated, enter the cell cycle, and then proliferate and turn into myoblasts, which are then differentiated into myocytes. The myocytes are fused and form myotubes, which finally turn into mature myofiber, the basic unit of skeletal muscle [78,79]. Although this proportion of activated SCs replenish myofibers, other SCs self-renew and return to their niche to maintain a normal number of SCs.

Several transcription factors are involved in the precise regulation of SC quiescence and activation; therefore, each transcription factor can be used as a marker for detecting the SC process. Paired box 7 (Pax7) is known as a canonical marker of SC. Quiescent SC expresses high levels of Pax7 but not myogenic regulating factors (MRFs). When activated, SCs lose expression of Pax7 and express MRFs. In each step of myogenesis, different types of MRF expressions occur. When SCs turn into myoblasts, myogenic differentiation 1 protein (MyoD) and myogenic factor 5 (Myf5) are expressed. Subsequently, when myoblasts are differentiated into myocytes, both myogenin and muscle-specific regulatory factor 4 (Mrf4) are expressed. In the final mature myofiber, MHC is expressed [79].

Aging induces a decline in the regenerative capacity of SCs through numerical and functional changes. A significantly reduced number of SCs, particularly in type II muscle fibers of the elderly (70–86 years of age) compared to that of younger adults (18–40 years of age) has been reported [80]. The functional loss of SCs in aging has been reported to result in cell-autonomous loss in self-renewal [81], a conversion from the myogenic lineage into the fibrogenic lineage [82], and the loss of quiescence [83]. Moreover, SC-dependent declines in muscle regenerative capacity in aging can affect sarcopenia [84]. For example, depletion of SCs accelerates age-related degeneration of neuromuscular junctions, a synapse located between motor neurons and skeletal muscle, and is one of the manifestations of sarcopenia [85].

## 3. Beneficial Effects of Natural Dietary Ingredients on Skeletal Muscle

Although substantial research on the prevention and/or treatment of sarcopenia have focused on exercise and diet which can restore and maintain muscle mass and function [86,87,88], an auxiliary therapy such as dietary supplementation can also help to prevent sarcopenia. Here, we focused various natural dietary ingredients, defined as their sources are found in nature that provides a potential health benefit [89,90]. They include botanical and marine extracts, phytochemicals, and probiotics and accumulating evidence has suggested that these ingredients can potentially reverse sarcopenia-related negative effects. In this section, recent studies published from 2018 to 2022 reporting the preventive functions of potential dietary substances against sarcopenia have been summarized according to the aforementioned pathological mechanisms including an imbalance between protein synthesis and degradation, aging-related inflammatory response, mitochondrial dysfunction, and SC dysregulation. Dietary substances-mediated improvements in sarcopenia diagnosis criteria and biochemical index assessing general health, muscle growth, and inflammatory status have also been explained. 

### 3.1. Improvement in Maintenance of Protein Homeostasis by Natural Dietary Ingredients

Various natural dietary ingredients such as botanical and marine extracts, phytochemicals, and probiotics have been found to regulate protein homeostasis through the PI3K/Akt pathway, UPS, and autophagy (Table 1).

The PI3K/Akt pathway is an important signaling pathway not only for the maintenance of protein homeostasis by enhancing muscle protein synthesis [27] but also for the process of skeletal muscle formation, myogenesis, which is mediated by MRFs such as myogenin, MyoD, and Myf5 [42].

Activation of myogenesis was reported in two plant-derived extracts from green tea and a mixture of three different plants (*Withania somnifera*, *Silybum marianum*, and *Trigonella foenum-graecum*; WST) and one phytochemical, isobavachalcone. Hong et al. reported that tannase-converted green tea extracts upregulated the expression of MRFs such as MyoD and myogenin in the biceps femoris of aged mice compared with those of the control young mice. Tannase-converted green tea extracts also increased protein levels of not only mTOR and p70S6K but also follistatin, an antagonist of the myostatin receptor, which induces skeletal muscle hypertrophy with the activation of the Akt/mTOR/p70S6K signaling cascade [100] in the biceps femoris of aged mice [91]. Salvadori et al. demonstrated that a herbal formulation WST activated the p38 MAPK/myogenin pathway in C2C12 myotubes in three different atrophic/sarcopenic models induced by the treatment of TNF-α/interferon γ, dexamethasone (Dex), or phosphate-buffered saline. C2C12 myotubes with atrophic/sarcopenic condition, vastus lateralis muscle biopsies with sarcopenia showed increase in myotube diameter, the number of nuclei inside the myotube, and MHC expression in WST-treated group through activation of the Akt pathway, specifically in the C2C12 model [92]. Hur et al. showed that isobavachalcone, a chalcone flavonoid that was originally isolated from *Psoralea corylifolia* (babchi), also upregulated the protein levels of MRFs, including myogenin and MyoD, in TNF-α-treated C2C12 myotubes compared to those in control myotubes. Simultaneously, isobavachalcone prevented the deleterious effect of TNF-α on myotube differentiation, as determined by the increase in myotube diameters and expression of MHC and myogenin proteins [96].

Promoting protein synthesis via activation of the PI3K/Akt pathway was reported in three plant-derived extracts from *Leonurus japonicus*, *Chrysanthemum morifolium* Ramat, and oligonol and four phytochemicals including leonurine, isochlrogenic acid A, corylifol A, and 5,7-dimethoxyflavone (DMF). Extracts from *Leonurus japonicus* and *Chrysanthemum morifolium* Ramat as well as their major active compounds such as leonurine (4-guanidino-*n*-butyl-syringate) and isochlorogenic acid A activated the PI3K/Akt pathway and enhanced protein synthesis in TNF-α-treated L6 myotubes. The PI3K/Akt pathway activation was validated by elevated protein phosphorylation levels of PI3K, Akt, mTOR, p70S6K, and/or 4E-BP1 in dietary component-treated myotubes compared to those in non-treated myotubes [93,94]. Activation of the PI3K/Akt/mTOR signaling pathway was also observed by treatment with oligonol, a flavanol-rich lychee extract, in the skeletal muscle of SAMP8 mice. Additionally, oligonol increased skeletal muscle mass, cross-sectional area (CSA), and grip strength in SAMP8 mice compared with those in non-treated SAMP8 mice [95]. To measure grip strength, mouse placed on the grid was pulled by its tail with increasing force until it was unable to grasp the grid. The maximal force exerted by the mouse was measured by a commercial grip strength meter and defined its grip strength. Two other phytochemicals, including corylifol A and DMF, also activated the PI3K/Akt signaling pathway in in vitro and in vivo models, respectively. Han et al. showed that corylifol A, a flavonoid from *Psoralea corylifolia* L., recovered Dex-induced downregulation of MHC and *p*-Akt protein levels. Corylifol A also protected against Dex-induced myotube loss by upregulating the proportion of multinucleated MHC-expressing myotubes [97]. Kim and Hwang showed that DMF, a major flavone from *Kaempferia parviflflora*, activated the PI3K/Akt and mTOR pathway by increasing the phosphorylation levels of PI3K, Akt, mTOR, p70S6K, and 4E-BP1 in aged mice. DMF also enhanced muscle weights (gastrocnemius, tibialis anterior, extensor digitorum longus, and soleus), the ratio of hindlimb muscle volume to body weight, and the CSA of the gastrocnemius muscle fiber compared to those of the non-treated control aged mice [101].

With regard to protein degradation, several natural dietary ingredients (four plant-derived extracts from green tea, *Leonurus japonicus*, *Chrysanthemum morifolium* Ramat., and oligonol, six phytochemicals including leonurine, isochlorogenic acid A, isobavachalcone, corylifol A, DMF, and carnosol, and one probiotic including *Lactobacillus plantarum* HY7715) have been revealed to inhibit UPS-mediated protein degradation, resulting in an increase in muscle mass. Hong et al. reported that tannase-converted green tea extracts decreased protein levels of *p*-FoxO3a and myostatin as well as those of MuRF1 and atrogin-1, two E3 ubiquitin ligases that are important regulators of ubiquitin-mediated protein degradation in the skeletal muscle, in the biceps femoris and/or soleus muscle of aged mice compared with those in the untreated mice [91]. Lee et al. reported that *Leonurus japonicus* extract and its major bioactive leonurine increased inactive *p*-FoxO3 protein level. *Leonurus japonicus* extract and leuonurine also decreased mRNA expressions of MuRF1 and atrogin-1 in TNF-α-treated L6 myotubes compared with those in non-treated myotubes [93]. Kwon et al. showed similar results by *Chrysanthemum morifolium* Ramat. extract and isochlorogenic acid A treatments, indicating a reduction in protein level of *p*-FoxO3a and mRNA levels of MuRF1 and atrogin-1 in TNF-α-treated L6 myotubes compared with those in non-treated myotubes [94]. Additionally, oligonol decreased protein levels of nuclear FoxO3 and mRNA levels of MuRF1 and atrogin-1 in the skeletal muscle of SAMP8 mice compared with those in non-treated mice [95]. Hur et al. showed that isobavachalcone reduced protein levels of muscle atrophy markers including *p*-FoxO1, atrogin-1, and MuRF1 in TNF-α-treated C2C12 myotubes compared with those in non-treated myotubes [96]. Han et al. reported that corylifol A decreased the gene expression levels of atrogin-1, MuRF1, and myostatin in Dex-treated C2C12 myotubes [97]. Kim and Hwang also reported that DMF decreased *p*-FoxO3 protein level and gene expression levels of MuRF1 and atrogin-1 in aged mice compared with those in non-treated aged mice [101]. Morel et al. showed that carnosol, the phenolic diterpene, suppressed both gene and protein expression levels of MuRF1 in human primary myotubes from healthy adults (19-, 30-, and 70-year-old adults) compared to with in non-treated counterpart myotubes. Muscle hypertrophy was also observed as shown by increase in myotube area and MHC protein expression [102]. Moreover, Lee et al. showed that *L. plantarum* HY7715 reduced mRNA levels of MuRF1 and atrogin-1 both in the soleus and gastrocnemius of aged mice, accompanied with an increase in muscle mass in soleus and gastrocnemius [99].

In addition, the effects of natural dietary ingredients including oligonol extract and two phytochemicals such as DMF and β-cryptoxanthin on autophagy, the other mechanism of protein degradation, has been also investigated as it is involved in muscle protein homeostasis. Chang et al. reported that oligonol decreased aging-induced elevated autophagy as shown by the decreased protein levels of Atg13, LC3-Ⅱ, and p62 in the skeletal muscle of SAMP8 mice compared with those in non-treated SAMP8 mice. Oligonol also restored abundant accumulation of autophagosomes and lysosomes, which indicates aging-induced impairment of autophagic flux, in the skeletal muscle of SAMP8 mice [95]. Kim and Hwang reported that DMF downregulated mRNA expression of autophagy-related proteins, including beclin1, LC3, Atg4, and Atg7, in DMF-treated aged mice compared with that in control aged mice [101]. Noguchi et al. demonstrated that β-cryptoxanthin, a provitamin A carotenoid, inhibited aging-induced increase in protein levels of autophagy-related factors such as beclin1, p62, LC3-I, and LC3-II in the soleus muscle of SAMP1 mice. The ratio of p62-positive fibers to p62-negative fibers in a CSA of the soleus muscle was also decreased in β-cryptoxanthin-administered SAMP1 mice compared with that in non-treated mice. Accordingly, muscle mass, CSA, and MHC type I protein levels in the soleus were increased in β-cryptoxanthin-treated SAMP1 mice compared with those in non-treated SAMP1 mice [98].

### 3.2. Protection against Inflammation by Natural Dietary Ingredients

The therapeutic effects of various natural dietary ingredients on inflammatory pathophysiology involved in sarcopenia are summarized in Table 2. Abolishment of SASP markers and the well-known pro-inflammatory NF-κB pathway have been proposed as strategic targets for therapeutic effects against sarcopenia-related inflammation phenotypes. Reduction of oxidative stress, which also increases with advancing age, was demonstrated as therapeutic target in some studies because oxidative stress and age are known to be interrelated as one can promote the other [59].

A reduction of pro-inflammatory cytokine levels was reported in two plant-derived extracts from ginseng and olive leaf and a phytochemical curcumin using in vitro or in vivo sarcopenic models. Oh et al. reported that the non-saponin fraction (NSF) of Korean red ginseng decreased the release of pro-inflammatory cytokines including IL-1, IL-6, TNF-α, and CRP in the serum of aged C57BL/6 mice compared with that in non-treated aged mice. NSF also restored serum levels of glutathione, which was lower in aged group compared to that in young group. The protein levels of other antioxidant enzymes including superoxide dismutase 1 (Sod1), glutathione peroxidase 1 (GPx1), and catalase were also increased after NSF treatment in the gastrocnemius and quadriceps femoris of aged mice [103]. Gonzalez-Hedstrom et al. demonstrated that olive leaf extract also reversed aging-induced upregulation of pro-inflammatory marker gene expressions of IL-6, IL-1β, and cyclooxygenase-2 in muscle tissue. On the contrary, aging-induced downregulated expression of anti-inflammatory cytokine IL-10 gene was increased in olive leaf extract-supplemented aged mice. Increased Gpx gene expression levels were also observed by olive leaf extract supplementation [104]. Liang et al. demonstrated that curcumin delivered a form of surface-modified hydroxyapatite (Cur-SHAP) by intramuscular route exerted anti-inflammatory and antioxidant effects in lipopolysaccharide (LPS)-treated C2C12 myotubes. The mRNA levels of IL-6, IL-1β, and TNF-α were decreased in the Cur-SHAP group following LPS induction compared with those in LPS-only group. The LPS-induced generation of reactive oxygen species (ROS) was also reduced to the control level by Cur-SHAP treatment [105].

Not only regulating the levels of pro-inflammatory cytokines but also modulating the target pathway, the NF-κB signaling pathway was reported in several dietary ingredients including two botanical and marine extracts, *Leonurus japonicus* extract and *Pyropia yezoensis* crude protein (PYCP), and four phytochemicals, namely, leonurine, isobavachalcone, quercetin, and diphlorethohydroxycarmalol (DPHC) in sarcopenic experimental models. *Leonurus japonicus* extract and its bioactive compound leonurine inhibited TNF-α-induced NF-κB protein expression in L6 rat myotubes. *Leonurus japonicus* extract and leonurine also inhibited the mRNA expression of IL-6 and TNF-α [93]. PYCP, isolated from an edible red alga in Southeast Asia, also inhibited the NF-κB signaling pathway as shown by the reduction in protein level of TNF-receptor1, translocation of NF-κB/p65, and cytosolic *p*-IkB-α in TNF-α-treated C2C12 myotubes. Furthermore, PYCP reduced IL-6 release in culture medium as well as intracellular ROS level in the serum [106]. The flavonoid isobavachalcone also showed anti-inflammatory effects by decreasing the translocation of phosphorylated-NF-κB p65 from the cytosol to nucleus in TNF-α-treated C2C12 myotubes. Activation of Nrf2, the key regulator of response to oxidative stress, and upregulated expression of its downstream antioxidant enzyme heme oxygenase-1 were also observed by isobavachalcone treatment [96]. Quercetin (3,3,4,5,7-pentahydroxyflavone), the other flavonoid abundant in various vegetables and fruits such as onions, tomatoes, and apples, suppressed IκB-α protein degradation, indicating a decrease in NF-κB activity in TNF-α-treated C2C12 myotubes [107]. DPHC, a phlorotannin isolated from the brown algae—*Ishige okamurae*—also exerted anti-inflammatory action with downregulation of NF-κB signaling as shown by reduced *p*-IκB and *p*-NF-κB protein expression levels. Increased production of nitric oxide and gene expression of pro-inflammatory cytokines including IL-6, IL-1β, and TNF-α were all reversed by DPHC treatment in TNF-treated C2C12 myotubes [108].

**Table 2:** Summary of studies reporting the role of natural dietary ingredients involved in inflammation improvement in sarcopenia prevention.

Natural Dietary Ingredients	Experimental Model	Experimental Designs	Results ^1^ (by Natural Dietary Ingredient Treatment)	Related Mechanisms (Potential Pathway)	Ref.
Botanical and marine extracts (bioactive compound)					
Non-saponin faction of Korean red ginseng	C57BL/6J mice	- young mice (3–6-month-old) - aged mice (20–24-month-old) - aged mice with ginseng (for 6 weeks)	↓ CRP, IL-1, IL-6, TNF-α ↑ GSH ↑ Catalase, GPx, Sod1	↓ inflammation ↓ oxidative stress	[103]
Olive leaf extract	Wistar rats	- young rats (3-month-old) - aged rats (24-month-old) - aged rats with olive leaf extract (for 3 weeks)	↓ *COX-2*, *IL-1β, IL-6* ↑ *IL-10* ↑ *GPx*	↓ inflammation ↓ oxidative stress	[104]
*Leonurus japonicus* extract (leonurine)	L6 myotube	- Non-treatment - TNF-α and *L. japonicus* extract or leonurine	↓ *IL-6*, *TNF-α* ↓ NF-κB	↓ inflammation (NF-κB pathway)	[93]
*Pyropia yezoensis* crude protein	C2C12 myotube	- Non-treatment - TNF-α - TNF-α and pyropia yezoensis protein	↓ IL-6 ↓ TNF-R1, NF-κB, *p*-IκBα ↓ intracellular ROS level	↓ inflammation (NF-κB pathway) ↓ oxidative stress	[106]
Phytochemicals					
Cur-SHAP	C2C12 myotube	- Non-treatment - LPS - LPS and Cur-SHAP	↓ *IL-6*, *TNF-α* ↓ ROS generation	↓ inflammation ↓ oxidative stress	[105]
Isobavachalcone	C2C12 myotube	- Non-treatment - TNF-α - TNF-α and isobavachalcone	↓ nuclear *p*-NF-κB p65 ↑ cytosolic *p*-NF-κB p65 ↑ HO-1, nuclear Nrf2 ↓ cytosolic Nrf2	↓ inflammation (NF-κB pathway)	[96]
Quercetin	C2C12 myotube	- Non-treatment - TNF-α - TNF-α and quercetin	↓ IκB-α	↓ inflammation (NF-κB pathway)	[107]
Diphlorethohydroxycarmalol	C2C12 myotube	- Non-treatment - TNF-α - TNF-α and diphlorethohydroxycarmalol	↓ NO production ↓ *IL-1β, IL-6*, *TNF-α* ↓ *p*-IκB-α, *p*-NF-κB p65	↓ inflammation (NF-κB pathway)	[108]
Probiotics					
*Lactobacillus casei* Shirota	SAMP8 mice	- young mice (16-week-old) - aged mice (28-week-old) - aged mice with *L. casei* Shirota (for 12 weeks)	↓ *TNF-α* ↑ *IL-10* ↓ protein carbonyl content	↓ inflammation ↓ oxidative stress	[109]
*Lactobacillus paracasei* PS23	SAMP8 mice	- young mice (16-week-old) - aged mice (28-week-old) - aged mice with *L. paracasei* PS23 (for 12 weeks)	↓ IL-6, MCP-1, TNF-α ↑ IL-10 ↓ protein carbonyl content ↑ *GPx, Sod*	↓ inflammation ↓ oxidative stress	[110]

^1^ ↑, increase; ↓, decrease. Name of target which expression was determined in gene level was italicized. Abbreviations: Cur-SHAP, curcumin loaded hydrophobic surface-modified hydroxyapatite; COX-2, cyclooxygenase-2; CRP, C-reactive protein; GPx, glutathione peroxidase; GSH, glutathione; HO-1, heme oxygenase-1; IκBα, inhibitor of nuclear factor-kappa B alpha; IL-1β, Interleukin-1 beta; IL-1, interleukin-1; IL-6, interleu-kin-6; IL-10, interleukin-10; LPS, lipopolysaccharide; MCP-1, monocyte chemoattractant protein-1; NF-κB, nuclear factor kappa B; Nrf2, nuclear factor erythroid-2-related factor 2; ROS, reactive oxygen species; SAMP, senescence-accelerated mouse prone; Sod, superoxide dismutase; TNF-α, tumor necrosis factor-alpha; TNF-R1, tumor necrosis factor-receptor1.

Two probiotics, namely, *Lactobacillus casei* Shirota (LcS) and *Lactobacillus paracasei* PS23 (LPP23) have anti-inflammatory therapeutic effects on aged muscle. LcS, historically (since 1930) known as human health-promoting probiotic, reduced pro-inflammatory cytokine TNF-α level and increased anti-inflammatory cytokine IL-10 level in the muscle of aged SAMP8 mice. Similar patterns of TNF-α and IL-10 levels were also observed in the serum of the mice, indicating that the anti-inflammatory effect of LcS was observed not only in the skeletal muscle but also in the whole-body system. LcS also displayed anti-oxidative effects as shown by the reduction of protein carbonyl content in muscle [109]. LPP23, a probiotic isolated from healthy human feces, exhibited downregulation of inflammation-related cytokines including IL-6, TNF-α, and MCP-1 in the serum of SAMP8 aged mice. Conversely, the serum and muscle expression levels of anti-inflammatory cytokine IL-10 were upregulated by LPP23 treatment. Decreased protein carbonyl content and increased gene expression levels of antioxidant enzymes, Sod and GPx, were also observed in the muscle of SAMP8 mice [110].

### 3.3. Enhancement of Mitochondrial Function by Natural Dietary Ingredients

The beneficial effects of various natural dietary ingredients on the regulation of mitochondrial biogenesis and dynamics, the common route of mitochondrial quality control [67], have been summarized in Table 3.

The majority of the studies investigated the impact of dietary ingredients on mitochondrial biogenesis improvement. Two plant-derived extracts from ginseng and green tea and three phytochemicals including ginsenoside, DMF, and fucoxanthin were investigated. Lee et al. reported that ginseng extract enriched by Rg3 and Rh2, named BST204, upregulated the gene expression levels and activity of Pgc-1α in TNF-α-treated C2C12 myotubes. The gene and protein expression levels of Nrf1 and Tfam, mitochondrial biogenesis-associated transcription factors, were also upregulated by BST204 treatment. BST204 also restored TNF-α-induced decrease in mitochondrial membrane potential (MMP), an index of ATP synthesis, accompanied with the upregulation of ATP content in myotubes [111]. The improvement of mitochondrial biogenesis by BST204 was also demonstrated in Dex-treated C2C12 myotubes, the other cell model of sarcopenia [112]. Ginsenoside Rg3 was also reported to have beneficial effects on mitochondrial biogenesis as shown by the increase in ATP content, activity and expression of Pgc-1α, and gene/protein expression levels of Nrf1 and Tfam in TNF-α-treated C2C12 myotubes [113]. Gras et al. showed that catechin-enriched green tea extract supplementation resulted in higher levels of Pgc-1α protein in the tibialis anterior and soleus muscles of aged mice compared with those of non-treated aged mice. The tibialis anterior of mice fed catechin-enriched green tea extract-supplemented diets also exhibited higher mitochondrial contents than those of controls, as shown by increased immunoreactivity level of ATP5A, a mitochondrial marker [114]. Kim et al. demonstrated that DMF rescued aging-induced reduction of mitochondrial biogenesis. DMF increased gene expression of Pgc-1α, Nrf-1, and Tfam in the soleus muscle of aged mice compared with those of non-treated aged mice. Consistently, the decrease in the relative mtDNA content in the aged group was also recovered by a DMF treatment [101]. Zhiyin et al. showed that fucoxanthin, a carotenoid found in brown seaweeds promoted mitochondrial biogenesis in Dex-treated C2C12 myotubes. Activating the sirtuin 1 (Sirt1)-related signaling pathway has been proposed as the mechanism involved in fucoxanthin enhancement of mitochondrial biogenesis given that fucoxanthin decreased the protein level of acetylated-Pgc-1α (inactive form) and Sirt1 inhibitor treatment offsets all effects of fucoxanthin [115]. 

**Table 3:** Summary of studies reporting the role of natural dietary ingredients involved in mitochondria function enhancement in sarcopenia prevention.

Natural Dietary Ingredients	Experimental Model	Experimental Designs	Results ^1^ (by Natural Dietary Ingredient Treatment)	Related Mechanism (Potential Pathway)	Ref.
Botanical and marine extracts (bioactive compound)					
BST204	C2C12 myotube	- Non-treatment - TNF-α (or Dex) - TNF-α (or Dex) and BST204	↑ *Nrf1*, *Pgc-1α*, *Tfam* ↑ Pgc-1α activity ↑ mitochondrial ATP content, MMP	↑ mitochondrial biogenesis	[111,112]
Catechin-enriched green tea extract	C57BL/6JRj mice	- young mice (4-month-old) - aged mice (22-month-old) - aged mice with GTE (for 6 month)	↑ Pgc-1α ↑ ATP5A	↑ mitochondrial content	[114]
Oligonol	SAMP8 mice	- young mice (SAMR1) - aged mice (32-week-old) - aged mice with oligonol (for 8 weeks)	↑ Ndufs8, Pgc-1α, Tfam ↑ mtDNA/nuclear DNA ratio ↑ Fis1, Mff, Mfn2, Opa1, PINK1 ↓ mitochondrial morphological changes (aberrance, swelling)	↑ mitochondrial biogenesis ↑ mitochondrial dynamics	[95]
*Ricinus communis* L. leaves extract (rutin)	C2C12 myotube	- Non-treatment - Dex - Dex and *R. communis* L. leaves extract or rutin	↑ mitochondrial OCR	↑ mitochondrial respiratory capacity	[116]
*Chrysanthemum zawadskii* Herbich extract (Acacetin-7-O-β-D-rutinoside)	C2C12 myotube	- Non-treatment - Dex - Dex and *C. zawadskii* Herbich extract or Aca-ce-tin-7-O-β-D-rutinoside	↑ mitochondrial OCR	↑ mitochondrial respiratory capacity	[117]
Phytochemicals					
Rg3	C2C12 myotube	- Non-treatment - TNF-α - TNF-α and Rg3	↑ Nrf1, Pgc-1α, Tfam ↑ mitochondrial ATP content, MMP	↑ mitochondrial biogenesis	[113]
5,7-Dimethoxyflavone (DMF)	C57BL/6J mice	- young mice (11-week-old) - aged mice (19-month-old) - aged mice with DMF (for 8 weeks)	↑ *Nrf1*, *Pgc-1α*, *Tfam* ↑ mtDNA content	↑ mitochondrial biogenesis	[101]
Fucoxanthin	C2C12 myotube	- Non-treatment - Dex - Dex and fucoxanthin	↑ Nrf1, Pgc-1α, Tfam ↓ acetylated Pgc-1α ↑ mitochondrial contents ↑ mitochondrial ATP content	↑ mitochondrial biogenesis (Sirt1/Pgc-1α pathway)	[115]
Dihydromyricetin	L6 myotube	- Non-treatment - Dex - Dex and dihydromyricetin	↑ mtDNA, Pgc-1α, Tfam ↓ mitochondrial morphological abnormality ↑ Mfn2, Tom20 ↓ change of MMP ↑ complex I and IV activity	↑ mitochondrial biogenesis ↑ mitochondrial dynamics ↑ mitochondrial respiratory capacity	[118]
Rg3	C2C12 myotube	- Non-treatment - Dex - Dex and Rg3	↑ mitochondrial OCR, ATP content, MMP ↓ mitochondrial morphological changes (swelling, increased volume) ↓ nuclear FoxO3, *p*-AMPK ↑ cytosolic FoxO3	↑ mitochondrial respiratory capacity (↓ AMPK/FoxO3 signaling pathway)	[119]
Probiotics					
*Lactobacillus paracasei* PS23	SAMP8 mice	- non-aged mice (16-week-old) - aged mice (28-week-old) - aged mice with *L. paracasei* PS23 (for 12 weeks)	↑ *Nrf1*, *Pgc-1a*, *Tfam* ↑ mtDNA copy number	↑ mitochondrial biogenesis	[110]
*Lactobacillus casei* Shirota	SAMP8 mice	- non-aged mice (16-week-old) - aged mice (28-week-old) - aged mice with *L*. *casei* Shirota (for 12 weeks)	↑ Sirt1, Tfam ↑ mtDNA copy number ↑ OCR	↑ mitochondrial biogenesis ↑ mitochondrial respiratory capacity	[109]

^1^ ↑, increase; ↓, decrease. Name of target which expression was determined in gene level was italicized. Abbreviations: AMPK, phosphorylated adenosine monophosphate-activated protein kinase; ATP, adenosine triphosphate; ATP5A, ATP synthase subunit alpha; BST204, a Rg3 and Rh2 enriched ginseng extract; Dex, dexamethasone; Fis1, mitochondrial fission protein 1; FoxO3, forkhead box O3; GTE, green tea extract; Mff, mitochondrial fission factor; Mfn2, miofusin 2; MMP, mitochondrial membrane potential; mtDNA, mitochondrial DNA; Ndufs8, NADH:ubiquinone oxidoreductase core subunit S8; OCR, oxygen consumption rate; Opa1, optic atrophy-1; Pgc-1α, peroxisome proliferator-activated receptor gamma coactivator 1-alpha; PINK1, PTEN-induced kinase 1; SAMP, senescence-accelerated mouse prone; Sirt1, Sirtuin 1; Tfam, mitochondrial transcription factor A; TNF-α, tumor necrosis factor-alpha; Tom20, translocase of the outer membrane of mitochondria 20.

Two studies of oligonol and dihydromyricetin have reported the impact of these dietary ingredients on the improvement of mitochondrial dynamics in a sarcopenia model. In the first study, the beneficial effects of oligonol on mitochondrial quality were investigated by studying the regulation of mitochondrial dynamics and biogenesis in SAMP8 mice. Oligonol improved mitochondrial fusion/fission dynamic by upregulating both mitochondrial fusion genes (*Mfn2* and *Opa1*) and mitochondrial fission genes (*Fis1* and *Mff*) compared with those in non-treated SAMP8 mice. Oligonol also upregulated the gene and protein expression of Pgc-1α, mRNA expression of *Tfam* and oxidative respiratory gene *Ndufs8*, and mtDNA/nuclear DNA ratio in SAMP8 mice. In addition, the protein level of PTEN-induced kinase 1 (PINK1), a gatekeeper of mitochondrial quality control, was increased in the oligonol-treated group. The improvement of derangements in mitochondrial dynamics by oligonol were also demonstrated by the restoration of the increase in aberrant and swollen mitochondria in the skeletal muscle [95]. Dihydromyricetin, a main flavonoid component of *Ampelopsis grossedentata* (vine tea), improved mitochondrial dynamics demonstrated by increased protein levels of Mfn2 in Dex-treated L6 myotubes. Dihydromyricetin also stimulated mitochondrial biogenesis as shown by increased protein level of not only Pgc-1α and Tfam but also Tom 20. Dihydromyricetin also attenuated Dex-induced changes including impairment of mitochondrial morphology and reduction of mtDNA content and MMP. Moreover, dihydromyricetin-induced enhancement of mitochondrial respiration capacity, a basic mitochondrial function, was observed as shown by the restoration of Dex-induced decrease in the activities of complex I and IV [118].

An enhanced mitochondrial function via respiratory capacity was reported in two plant-derived extracts from *Ricinus communis* L. leaf extracts and *Chrysanthemum zawadskii* Herbich (CZH) and three phytochemicals including rutin, acacetin-7-O-β-D-rutinoside, and ginsenoside Rg3. *Ricinus communis* L. leaf extracts (RC) and its bioactive compound rutin increased mitochondrial respiration measured by mitochondrial oxygen consumption rate (OCR) in Dex-treated C2C12 myotubes. RC and rutin recovered mitochondrial respiration at the basal stage, ATP-linked, maximal respiration, and spare capacity as compared with those in the Dex-treated group [116]. CZH, used in traditional medicine to treat inflammatory diseases and known as “Gujulcho” in Korea, and its active compound acacetin-7-O-β-D-rutinoside also exerted improvement in mitochondrial respiration function in Dex-treated C2C12 myotubes [117]. Lastly, the recovery of Dex-induced reduction in OCR, intracellular ATP levels, and MMP was observed in Rg3-treated C2C12 myotubes. The morphological changes including swelling and increased volume of mitochondria by Dex treatment was also rescued by Rg3 treatment. Inhibition of the AMPK/FoxO3 signaling pathway, represented by the reduction of AMPK phosphorylation and FoxO3 translocation into the nucleus, was proposed as a possible mechanism of protective action of Rg3 [119].

In addition, two probiotics including LPP23 and LcS have been reported to have therapeutic effects on sarcopenia via mitochondrial function enhancement. LPP23, a probiotic isolated from healthy human feces, preserved an aging-induced decline of mitochondrial biogenesis in aged SAMP8 mice. LPP23 increased the expression levels of mitochondrial biogenesis-associated genes including Pgc-1α, Nrf-1, and Tfam. LPP23 also upregulated mtDNA copy number [110]. LcS, the other well-known probiotic, also improved aging-induced dysregulation of mitochondrial biogenesis. LcS increased the gene expressions of Sirt1 and Tfam as well as mtDNA number in aged SAMP8 mice compared with those in non-treated mice. Consistently, OCR values of the LcS-supplemented SAMP8 mice were higher than those of non-supplemented SAMP8 mice [109]. 

### 3.4. Reversal of SC Dysfunction by Natural Dietary Ingredients

Two plant-derived extracts from Lemon myrtle and green tea and four phytochemicals including casuarinin, cocoa flavanols, curcumin, and sinensetin were reported to combat sarcopenia by increasing SC population and activation as summarized in Table 4.

Lemon myrtle (*Backhousia citriodora*) extract and its active compound casuarinin, one of hydrolysable tannins, activated SCs as shown by increased BrdU incorporation, an indicator of cell activation, in isolated SCs from muscle in 13-week-old SD rats [120]. Catechin-rich green tea extract supplementation increased densities of SCs as represented by higher percentages of Pax7-staining SCs in the tibialis anterior and soleus muscles from aged mice compared with those from non-treated aged mice [114]. In the same study, comparable beneficial effects of cocoa flavanols on SC density was also demonstrated with the same biomarkers as those tested for green tea extract [114]. Supplementation of the other dietary polyphenol, curcumin, also improved SC commitment and recruitment in 18-month-old C57BL/10ScSn mice. Curcumin maintained adult levels of myofiber maturation in soleus muscle regeneration after acute damage and increased the proportion of MyoD-positive SCs in the hindlimb muscles [121]. Lastly, sinensetin, a citrus-methylated flavone, increased differentiation rate of SCs isolated from thigh and calf muscle cells from 12-month-old SD rats. The primary SCs from sinensetin-treated group displayed longer diameter of myoblasts and higher protein expression of MRFs, including MyoD and myogenin, compared with those from non-treated group [122].

**Table 4:** Summary of studies reporting the role of natural dietary ingredients involved in satellite cell regulation in sarcopenia prevention.

Natural Dietary Ingredients	Experimental Model	Experimental Design	Result ^1^ (by Natural Dietary Ingredient Treatment)	Related Mechanism (Potential Pathway)	Ref.
Botanical and marine extracts (bioactive compound)					
Lemon myrtle extract (casuarinin)	SD rats	- young rats (13-week-old) - young rats with Lemon myrtle extract or casuarinin (for 4 days)	↑ BrdU incorporation into SCs	↑ SC activation	[120]
Catechin-rich-green tea extract	C57BL/6JRj mice	- young mice (4-month-old) - aged mice (22-month-old) - aged mice with GTE (for 6 month)	↑ Pax7-positive SCs	↑ SC population	[114]
Phytochemicals					
Cocoa flavanols	C57BL/6JRj mice	- young mice (4-month-old) - aged mice (22-month-old) - aged mice with cocoa flavanols (for 6 month)	↑ Pax7-positive SCs	↑ SC population	[114]
Curcumin	C57BL/10ScSn mice	- young mice (6-month-old) - aged mice (18-month-old) - aged mice with curcumin (for 6 months)	↑ myofiber regeneration after acute damage ↑ MyoD-positive SC	↑ SC activation	[121]
Sinensetin	Isolated SCs from SD rats	- young rats (6-week-old) - aged rats (24-month-old) - aged rats with sinensetin (for 5 days)	↑ myoblast differentiation of SC ↑ MyoD, myogenin	↑ SC activation	[122]

^1^ ↑, increase. Abbreviations: BrdU, bromodeoxyuridine; GTE, green tea extract; MyoD, myogenic differentiation protein 1; Pax7, paired box 7; SC, satellite cell.

### 3.5. Improvement in Diagnosis Criteria and Biochemical Index for Sarcopenia by Natural Dietary Ingredients

Sarcopenia is diagnosed by the decline of three main factors, namely, muscle mass, muscle strength, and physical performance. There were several studies testing the improvement of these criteria by dietary components. These dietary components comprise four of botanical and marine extracts, namely, tannase-treated green tea extract, fermented sarco oysters extract, shatavari, and *Ishige okamurae* extract; two phytochemicals, DPHC and curcumin; and two probiotics from *L. plantarum*. The studies were double-blind RCT with human participants or experimental animal studies with rodents. Changes of biochemical index such as inflammatory cytokines, growth factors, and stress hormones were also determined in these studies (Table 5). The exact number of participants and the amount and duration of supplementation in RCT are provided in the table.

Seo et al. showed that the tannase-treated green tea extract containing a high content of higher bioavailable compounds such as epicatechin and gallic acid exerted an improvement in muscle strength but not in muscle mass after a 12-week supplementation in older adults more than 60 years of age in a single-center RCT. An increase in muscle strength was determined by increased isokinetic flexor muscle (maximum muscle strength of the flexor muscle of the right leg) and handgrip strength in the treatment group compared with those in the placebo group. In a blood analysis, there was no difference in the levels of follistatin, which inhibits myostatin, CRP, IL-6, IL-8, IGF-1, and cortisol between the baseline and those after supplementation. On the contrary, the levels of myostatin, a negative regulator of muscle growth, were decreased after 12 weeks in the treatment group [123]. Rheu et al. revealed that the administration of fermented sarco oysters (*Crassostrea gigas*) extract for 12 weeks to postmenopausal women aged >65 years increased muscle strength as determined by muscle 60°/s right knee extension peak torque and grip strength. This improvement did not accompany changes in muscle mass and blood CRP levels. However, IGF-1 levels were increased in the treated group [124]. O’Leary et al. reported that supplementation of shatavari, an ayurvedic herb for women’s health, improved handgrip strength in 20 postmenopausal women (mean age, 68.5 years) after a 6-week supplementation [125]. Extract of *Ishige okamurae*, a brown alga, improved muscle mass and strength in an in vivo model of sarcopenia. In 14-month-old female mice, administration of *Ishige okamurae* extract or its active compound DPHC improved physical performance as shown by a decreased time for ladder climbing compared to that in the non-supplemented aged group. Serum levels of inflammatory cytokines including IL-1 β, IL-6, and TNF-α were also downregulated by supplementation [126].

Two studies reported of the beneficial role of curcumin in a specific form, which heightened its bioavailability against sarcopenia. Liang et al. reported that the administration of curcumin as Cur-SHAP via intramuscular injection improved muscle mass, strength, and physical performance in LPS-treated 12-month-old SD rats. In a serological analysis, no sign of chronic toxicity of Cur-SHAP was shown. Decreased levels of creatine kinase, lactate dehydrogenase, alanine aminotransferase, markers for muscle degradation, cell damage, and liver disease and general health were observed in Cur-SHAP-treated rats compared with those in only LPS-treated rats. No differences among all groups were observed in the levels of total protein and Ca^2+^, whose dysregulation is a common underlying phenomenon in the pathophysiology of muscles, including sarcopenia [105]. Varma et al. also reported that Cureit, which has a 10-fold greater bioavailability than curcumin improved handgrip strength in healthy older adults >65 years of age [127].

As mentioned above, two studies have been reported in which the beneficial action of probiotics against sarcopenia have been described. Lee et al. reported that 18 week-supplementation of *L. plantarum* TWK10, isolated from Taiwanese pickled cabbage improved muscle mass, muscle strength, and physical performance in older adults aged between 55 and 85 years, with mild fragility. The left-hand grip strength in the TWK10 high-dose group was higher than that in the baseline strength. The gait and balance ability were also improved as shown by a decreased test time in a 3-m timed up and go test after TWK10 supplementation. Walking ability, lower-limb muscle strength, and endurance were also improved as determined by a decreased time for the 10-m walk test and an increased time for the 30 s chair stand test after supplementation [128]. The other probiotics, *L. plantarum* HY7715, isolated from kimchi, also improved muscle mass, muscle strength, and physical performance in an in vivo study. HY7715-treated aged mice increased forelimb and all-limb grip strength and displayed longer treadmill distance compared with those in the untreated aged mice. HY7715 treatment also improved physiological fatigue after treadmill exercise at 2-week intervals as shown by decreased plasma levels of lactate, blood urea nitrogen, and creatine compared with those in the non-treated group [99]. 

## 4. Limitations

Natural dietary ingredients are potential candidates for the prevention and treatment of many diseases including sarcopenia, but their application in humans may be limited due to several reasons. Firstly, toxicity and safety issues should be considered. For example, there are reports on hepatotoxicity and gastrointestinal disorders, especially when green tea extracts are consumed on an empty stomach [129]. In the case of marine products, the risk of toxicity and adverse effects of small particles, such as microplastics and nanoplastics to human need to be deliberated [130]. Moreover, interactions of dietary ingredients with medications should also be carefully examined. This is particularly important since older adults are commonly taking multiple medications. An example is the interaction between ginseng and other drugs. Taking phenelzine and ginseng together causes tremor and headaches. Taking warfarin with ginseng lowers warfarin blood concentration and anticoagulation action [131]. Therefore, the risk of significant adverse events due to dietary ingredients and drug interactions should be carefully investigated. Finally, the low bioavailability and efficacy of dietary ingredients also limit the generalization of the experimental data to the human body directly [132]. An additional limitation is that the human data shown in Table 5 were also conducted with a very limited number of subjects. Table 5 summarized two animal studies to report the results of assessing all three diagnosis criteria including muscle mass, strength, and physical performance. However, most other in vivo studies presented in Table 1 did not examine the diagnosis criteria specifically in terms of physical performance. This indicates that further studies focusing on mechanistic validation in animals will also need to demonstrate changes in criteria to clarify that sarcopenia can be alleviated by a specific treatment.

## 5. Conclusions

Natural dietary ingredients are of great interest in the prevention and management of sarcopenia. This review showed multiple lines of evidence that various dietary materials including botanical/marine extracts, phytochemicals, and probiotics exert preventative and therapeutic effects against sarcopenia through a variety of mechanisms. These dietary ingredients recover protein homeostasis imbalance, chronic low-level inflammation, mitochondrial dysfunction, and SC dysfunction that can be induced in aged muscles. Despite these positive impacts of dietary ingredients on sarcopenia, potential drawbacks, such as safety/toxicity, drug interaction, and low bioavailability should also be considered. Based on both the pros and cons of a dietary approach for the prevention and management of sarcopenia, effective and low-risk clinical practices are required for developing dietary supplements against sarcopenia.

## Figures and Tables

**Table 1:** Summary of studies reporting the role of natural dietary ingredients involved in the maintenance of protein homeostasis in sarcopenia prevention.

Natural Dietary Ingredients	Experimental Model	Experimental Designs	Results ^1^ (by Natural Dietary Ingredient Treatment)	Related Mechanisms (Potential Pathway)	Ref.
Botanical and marine extracts (bioactive compound)					
Tannase-converted green tea extract	ICR mice	- aged mice (24-month-old) - aged mice with tannase-converted green tea extract	↑ *MyoD, myogenin* ↓ *p*-FoxO3a, myostatin, MuRF1, atrogin-1 ↑ lean mass	↑ myogenesis ↓ protein degradation (UPS; FoxO3 pathway)	[91]
WST	C2C12 myotube	- PBS and WST - TNF-α/IFNγ and WST - Dex and WST	↑ myogenin, *p*-p38 ↑ myotube diameter ↑ the number of nuclei inside myotube, total nuclei ↑ MHC type II, *p*-Akt	↑ myogenesis (p38 MAPK/myogenin pathway) ↑ protein synthesis (PI3K/Akt pathway)	[92]
	Human (Vastus lateralis muscle biopsies)	- young subjects - sarcopenic subjects	↑ myotube diameter ↑ the number of nuclei inside myotube ↑ MHC expression	↑ myogenesis	
*Leonurus japonicus* extract (leonurine)	L6 myotube	- Non-treatment - TNF-α and *L. japonicus* extract or leonurine	↑ *p*-PI3K, *p*-Akt, *p*-mTOR, *p*-p70S6K, *p*-4E-BP1 ↑ *p*-FoxO3 ↓ *atrogin-1*, *MuRF1* ↑ myotube diameter	↑ protein synthesis (PI3K/Akt pathway) ↓ protein degradation (UPS; FoxO3 pathway)	[93]
*Chrysanthemum morifolium* Ramat. extract (isochlorogenic acid A)	L6 myotube	- Non-treatment - TNF-α - TNF-α and *C. morifolium* Ramat. extract or isochlorogenic acid A	↑ *p*-PI3K, *p*-Akt, *p*-mTOR, *p*-p70S6K, *p*-4E-BP1 ↓ *p*-FoxO3 ↓ *atrogin-1*, *MuRF1*	↑ protein synthesis (PI3K/Akt pathway) ↓ protein degradation (UPS; FoxO3 pathway)	[94]
Oligonol	SAMP8 mice	- SAMR1 control mice (32-week-old) - SAMP8 mice (32-week-old) - SAMP8 mice with oligonol (for 8 weeks)	↑ *p*-Akt, *p*-mTOR, *p*-p70S6K ↓ nuclear localization of FoxO3a and NF-κB ↓ *atrogin-1*, *MuRF1* ↓ Atg13, LC3-II, p62 ↑ CSA, grip strength, muscle mass	↑ protein synthesis (PI3K/Akt pathway) ↓ protein degradation (UPS; FoxO3 pathway, autophagy)	[95]
Phytochemicals					
Isobavachalcone	C2C12 myotube	- Non-treatment - TNF-α - TNF-α and isobavachalcone	↑ myogenin, MyoD, MHC, myotube diameter ↓ atrogin-1, MuRF1, *p*-FoxO1	↑ myogenesis ↓ protein degradation (UPS)	[96]
Corylifol A	C2C12 myotube	- Non-treatment - Dex - Dex and corylifol A	↑ *p*-Akt, MHC ↓ atrogin-1, MuRF1, myostatin ↑ number of multinucleated myotube	↑ protein synthesis ↓ protein degradation (UPS)	[97]
β-Cryptoxanthin	SAMP1 mice	- SAMR1 control mice (20-week-old) - SAMP1 mice (20-week-old) - SAMP1 mice with β-Cryptoxanthin (for 15 weeks)	↓ beclin1, p62, LC3-I, LC3-II ↑ mass, CSA, MHC type I (soleus)	↓ protein degradation (autophagy) ↑ muscle hypertrophy	[98]
Probiotics					
*Lactobacillus plantarum* HY7715	Balb/c mice	- young mice (7-week-old) - aged mice (81-week-old) - aged mice with HY7715 (for 5 weeks)	↓ *atrogin-1, MuRF1* ↑ mass (soleus, gastrocnemius) ↑ CSA, *MyoD*, *MHC* type I	↓ protein degradation (UPS) ↑ muscle hypertrophy	[99]

^1^ ↑, increase; ↓, decrease. Name of target which expression was determined in gene level was italicized. Abbreviations: Akt, protein kinase B; Atg, autophagy-related; CSA, cross-sectional area; Dex, dexamethasone; FoxO3, forkhead box O3; IFNγ, interferon gamma; LC3, light chain 3; MAPK, mitogen-activated protein kinase; MHC, myosin heavy chain; mTOR, mammalian target of rapamycin; MuRF1, muscle RING-finger protein-1; MyoD, myogenic differentiation 1 protein; NF-κB, nuclear factor kappa B; PI3K, phosphatidylinositol 3-kinase; p70S6K, ribosomal protein S6 kinase; SAMP, senescence-accelerated mouse prone; SAMR, senescence-accelerated mouse resistance; TNF-α, tumor necrosis factor-alpha; UPS, ubiquitin-proteasome system; WST, *Withania somnifera*, *Silybum marianum*, *Trigonella foenum-graecum* formulation; 4E-BP1, 4E-binding protein 1.

**Table 5:** Summary of studies reporting the role of natural dietary ingredients in improvements of criteria for sarcopenia and biochemical index related to sarcopenia.

Natural Dietary Ingredients	Experimental Model	Experimental Designs	Treatment	Criteria for Sarcopenia ^1^ (Mass/Strength/Physical Performance)		Biochemical Index ^2^	Ref.
Botanical and marine extracts (bioactive compound)							
Tannase-treated green tea extract	Older adults (>60 yr, 10 males and 57 females)	- placebo-control (*n* = 34) - Tannase-treated green tea extract (*n* = 33)	600 mg/d for 12 weeks	Mass	= body muscle mass	= follistatin, CRP, IL-6, IL-8, IGF-1, cortisol ↓ myostatin	[123]
				Strength	↑ isokinetic flexor strength (right leg), handgrip strength		
				Physical performance	ND		
Fermented sarco oysters extract	Postmenopausal women with low muscle mass (> 65 yr)	- placebo-control (*n* = 23) - Fermented sarco oysters extract (*n* = 23)	1000 mg/d for 12 weeks	Mass	= appendicular skeletal mass/height^2^	= CRP ↑ IGF-1	[124]
				Strength	↑ quadriceps muscle strength, handgrip strength		
				Physical performance	ND		
Shatavari	Postmenopausal women with low muscle mass (> 60 yr)	- placebo-control (*n* = 10) - Shatavari (*n* = 10)	1000 mg/d for 6 weeks	Mass	ND	ND	[125]
				Strength	↑ handgrip strength = knee extensor strength		
				Physical performance	ND		
*Ishige okamurae* (diphlorethohydroxycarmalol)	C57BL/6J mice	- young mice (4-month-old) - aged mice (14-month-old) - aged mice with *I. okamurae*	50–200 mg/kg/d for 7 weeks	Mass	↑ lean mass	↓ IL-1β, IL-6, TNF-α	[126]
				Strength	↑ grip strength		
				Physical performance	↓ the required time for ladder climbing		
Phytochemicals							
Cur-SHAP	SD rats	- control rats with saline (12-month-old) - sarcopenic rats with LPS - sarcopenic rats with LPS + Cur-SHAP	150 mg/kg/d 4 times for 8 weeks	Mass	↓ fat/lean mass ratio (%)	↓ creatine kinase, lactate dehydrogenase, ALT = total protein, calcium	[105]
				Strength	↑ grip strength		
				Physical performance	↑ muscle endurance by treadmill test		
Cureit	Older adults (>65 yr, 13 males and 17 females)	- placebo-control (*n* = 15) - Cureit (*n* = 15)	500 mg/d for 12 weeks	Mass	ND	ND	[127]
				Strength	↑ handgrip strength		
				Physical performance	ND		
Probiotics							
*Lactobacillus plantarum* TWK10	Older adults with frailty (55–85 yr, 25 males and 17 females)	- placebo-control (*n* = 17) - low-dose TWK10 (*n* = 12) - high-dose TWK10 (*n* = 13)	2 × 10^10^ or 13; 6 × 10^10^ CFU/day for 18 weeks	Mass	↑ muscle mass	ND	[128]
				Strength	↑ handgrip strength		
				Physical performance	↑ gait and balance ability ↑ the walking ability, lower-limb muscle strength, and endurance		
*Lactobacillus plantarum* HY7715	Balb/c mice	- young mice (7-week-old) - aged mice (81-week-old) - aged mice with HY7715	1 × 10^8^ CFU/kg /day for 5 weeks	Mass	↑ muscle weight (soleus, gastrocnemius)	↓ lactate, BUN, creatinine = AST, ALT	[99]
				Strength	↑ grip strength (fore and all limb)		
				Physical performance	↑ running endurance (treadmill distance)		

^1,2^ ↑, increase; ↓, decrease; =, no change; ND, not determined. Abbreviations: ALT, alanine aminotransferase; AST, aspartate aminotransferase; BUN, blood urea nitrogen; CFU, colony forming unit; Cur-SHAP, curcumin loaded hydrophobic surface-modified hydroxyapatite; CRP, C-reactive protein; Cureit, the novel bioavailable curcumin; IGF-1, insulin-like growth factor 1; IL-1β, interleukin-1 beta; IL-6, interleukin-6; IL-8, interleukin-8; LPS, lipopolysaccharide; TNF-α, tumor necrosis factor-alpha.

## Data Availability

Not applicable.
